# Supplementary material for: WordCommentsAnalyzer: A windows software tool for qualitative research
Source: F1000Res. 2018 Sep 4;7:536. Originally published 2018 May 3. [Version 2] doi: 10.12688/f1000research.14819.2 (PMC6137411; doi:10.12688/f1000research.14819.2)
Supplement: Supplementary file 3 [file f1000research-7-17614-s0002.tgz › b2a79496-4ada-47c4-9682-a99ce867836d.zip!/Eight Iranian Health Journals/Archives of Rehabilitation_50.docx]

© Archives of Rehabilitation. This work is licensed under CC BY-NC 4.0 (https://creativecommons.org/licenses/by-nc/4.0/).

Volume & Issue: دوره 8 -

Title: سخن سردبیر

http://rehabilitationj.uswr.ac.ir/article-1-123-fa.html

چرا ویژه نامه اختلالات روانی؟ مسائل روانی زیربنای بیشتر بیماریهای جسمی و تقریباً همه آسیب‌های اجتماعی را تشکیل می‌دهد. فردی را در نظر بگیرید که در اثر تصادف رانندگی دچار شکستگی پاشده است. اینجا نخست باید پرسید چه عاملی باعث شد این فرد بدون احتیاط رانندگی کند؟ رانندگی بی‌توجه به امنیت خود و دیگران از ویژگیهای افراد ضد اجتماعی، مبتلایان به اختلال شخصیت مرزی، بیماران هیپومانیک و مانیک و بیماران افسرده شدید است که بویژه مورد آخر یعنی بیماران افسرده شدید ممکن است در کل تمایل به خودکشی آشکار یا پنهان داشته باشند. از سوی دیگر افرادی که در معرض عوامل استرس آور قرار می‌گیرند و دچار نابسامانی سازگاری می‌شوند هم زیاد در خطر تصادف قرار می‌گیرند، به نحوی که به این گروه نام «مستعد به تصادف » گذاشته‌اند. عدم تمرکز حواس منجر به تصادف رانندگی می‌تواند در اثر بیماریهای روانی کارکردی نظیر افسردگی، اضطراب، نابسامانی فشار روانی به دنبال سانحه، یا ناشی از بیماریهای روانی عضوی چون آلزهایمر باشد. کسانیکه الکل یا قرصهای محرک یا کوکایین مصرف می‌کنند نیز با میزانهای بالاتر تصادف رانندگی مواجه می‌شوند. مصرف این مواد نیز تحت تأثیر عوامل روانی است. اهمیت موضوع در این حد متوقف نمی‌ماند. واکنش‌های فرد به تصادف، شکستگی‌ها، نشانه‌های بیماری و معلولیت‌ها، رعایت یا عدم رعایت دستورهای پزشکی همه به مسائل روانی مربوط می‌شود. در دیگر وضعیت‌های پزشکی هم اوضاع به همین منوال است. زمانیکه از افزایش بیماریهای قلبی – عروقی، مثلاً بیماریهای شریان اکلیلی قلب، افزایش فشار خون شریانی، یا حتی سرطان – برای نمونه سرطان ریه – گفتگو می‌شود، بهتر است در ابتدا بپرسیم چه عامل یا نابسامانی روانی باعث شد بیمار سیگار بکشد، ورزش نکند، دچار اختلال خواب شود، غذای نامناسب بخورد و در نتیجه خود را در معرض بیماریهای گوناگون جسمی قرار دهد. آسیب‌های اجتماعی هم که این روزها زیاد در مورد آن صحبت می‌شود، نظیر بزهکاری، اعتیاد، خانواده‌های ازهم گسیخته، و ... در اثر مشکلات روانی تک تک افراد به وجود می‌آید. این درست است که عوامل اجتماعی می‌تواند مسائل مذکور را تشدید کند یا تخفیف دهد، اما زمینه تاحد زیادی در وجود خود فرد قرار دارد. برای نمونه، ما بزهکاری نداریم که صرفاً در اثر عوامل بزهکار شده باشد. بخش عمده بزهکاران را مبتلایان به نابسامانی شخصیت ضد اجتماعی تشکیل می‌دهند که اگر در محیط مناسب قرار گیرند، بزهکاری و قانون شکنی هم می‌کنند. بسیاری از موضوعاتی که سابق بر این به عنوان مسائل اجتماعی مطرح می‌شد، امروزه می‌دانیم که به طور عمده ناشی از بیماریهای روانی است. از این جمله باید به خودکشی اشاره کنیم. کاربرد صحیح و بجای داروهای نوین ضد افسردگی در دو دهه اخیر در کشورهای صنعتی غرب باعث شد میزان خودکشی در گروههای جوان و نوجوان تاحد چشمگیری پایین بیاید و این برخلاف روند کشورهای درحال توسعه است که در آنها آمار خودکشی در گروههای نوجوان و جوان رو به فزونی است، زیرا در این کشورها بیماریهای روانی مورد توجه جدی و درمان قرار نمی‌گیرد و از دارو استفاده لازم نمی‌شود. اگر برای پزشکان، مردم و مسئولان روشن شود که بیماریهای جسمی و آسیب‌های اجتماعی در واقع زمینه و علت روانی دارند، آنگاه مطمئناً روانپزشکی و رواندرمانی هم در اولویت‌های اول وزارت بهداشت، قوه قضاییه و وزارت کشور قرار می‌گرفت و جایگاه راستین خود را می‌یابد. هنوز هم اکثریت پزشکان می‌اندیشند بیمار واقعی کسی است که ضایعه بافتی قابل مشاهده داشته باشد. چه بسیارند بیمارانی که به علت مشکلات روانپزشکی تحت درمان هستند ولی بنا به توصیه نادرست سایر پزشکان و حتی برخی همکاران این حوزه که تحت عنوان مشاوره و رواندرمانی غیر دارویی بدون نظارت روانپزشک به فعالیت می‌پردازند، درمان خود را رها کرده‌اند. بسیاری از بیماران افسرده به دلیل همین توصیه‌های نادرست و قطع درمان، پس از مدتی در اثر شدت افسردگی دست به خودکشی زده‌اند. بیماری که هذیانهای او با مصرف داروهای روانپزشکی تحت کنترل در آمده، بدلیل قطع دارو متعاقب توصیه غلط پس از مدتی با برگشت هذیانهای خیانت زناشویی همسر خود را به قتل رسانیده است. هنوز اکثریت پزشکان توجه ندارند که مسائل روانپزشکی در انتهای بررسیهای پزشکی قرار ندارد بلکه جای آن از ابتدا و به موازات سایر بررسیها می‌باشد. اینگونه نیست که بیمار فقط از جنبه مشکلات جسمی بررسی شود و اگر چیزی در این باب یافت نشد به روانپزشک ارجاع شود. بلکه ارتوپد هم باید بداند چرا مراجع او بدون احتیاط قدم بر می‌دارد و چنان شتابزده می‌رود که جلوی پایش را نمی‌بیند، یا جراح باید بیندیشد چگونه این بیمار در عرض دو ماه سه بار تصادف منجر به جراحت داشته است، مشکل باز می‌گردد به میزان کم آموزش روانپزشکی در دوره پزشکی و تعامل کمتر تخصصهای مختلف درمانی و توانبخشی با روانپزشکان و سایر همکاران حوزه روانشناختی، رواندرمانی و روانپرستاری. در حال حاضر دانشجوی پزشکی در بیمارستانهای روانی یا به ندرت در بخش روانپزشکی در بیمارستان عمومی فقط با بیماران روانپریش روبرو می‌شود و این امر به او فرصت نمی‌دهد تا با اکثریت بیماران روانپزشکی که غیر روانپریش هستند و یا بیماران داخلی و جراحی و زنان و کودکان که مشکلات روانپزشکی دارند اما به درمانگاههای غیر روانپزشکی مراجعه می‌کنند آشنا شود. درحالیکه ضروری است در همه مجموعه‌های درمانگاهی اعم از داخلی، جراحی، پوست، قلب، گوارش، غدد، ... دستکم یک روانپزشک رابط حضور داشته باشد و در کنار متخصصان مربوطه به آموزش مسائل روانپزشکی همه بیماران به دانشجو و دستیار بپردازد. کشورما به تربیت تعداد زیادی روانپزشک در مدت بالنسبه کوتاه برای رسیدن به حداقل استانداردها در مورد نسبت روانپزشک به کل جمعیت نیاز دارد که طبق برآوردهای ارائه شده در جلسات کمیته تدوین برنامه راهبردی رشته تخصصی روانپزشکی، در حدود پانزده برابر تعداد کنونی روانپزشکان ایران است که در حال حاضر فقط یکهزار نفر هستند. همچنین سایر متخصصین در حوزه روانشناسی، رواندرمانی و روانپرستاری نیز «خصوصاً در مقطع دکترای تخصصی» تعداد شان کمتر از استانداردهای جهانی است. تقویت و گسترش گروههای روانپزشکی موجود، و تأسیس گروههای جدید آموزشی تخصصی روانپزشکی از دیگر مباحث طرح شده در کمیته تدوین برنامه راهبردی رشته تخصصی روانپزشکی است که شامل بهبود و گسترش کیفی این رشته علاوه بر گسترش کمی آن می‌باشد. گروه روانپزشکی دانشگاه علوم بهزیستی و توانبخشی با مرکزیت بیمارستان بزرگ آموزشی – درمانی روانپزشکی رازی در طول ده سال اخیر نقش مهمی در تربیت روانپزشکان آگاه و آشنا با شرایط واقعی بیماران کشور، و مطلع از اهمیت پیشگیری و توانبخشی، علاوه بردرمان داشته است به نحوی که از سایر دانشگاههای علوم پزشکی، دستیاران برای گذراندن دوره‌های عمومی یا فوق تخصصی (روانپزشکی کودک و نوجوان) و کارهای پژوهشی به مرکز روانپزشکی رازی مراجعه می‌کنند. با توجه به وجود مرکز روانپزشکی رازی با داشتن 1400 تخت بستری و درمانگاههای متعدد که نزدیک به 20% کل خدمات درمانی روانپزشکی کشور را ارائه می‌کند، امکان استفاده بیشتر آموزشی و پژوهشی در جهت نیل به اهداف وزارتخانه متبوع در زمینه روانپزشکی وجود دارد. این مرکز و متخصصین مختلف روانپزشک، روانشناس و ... نه تنها در تربیت سالانه 18 نفر پزشکان دستیار روانپزشکی و درمان بیماران و امور پژوهشی روانپزشکی به طور مستقیم به فعالیت مشغولند بلکه به صورت همکار در امور آموزشی و پژوهشی گروههای دیگر آموزشی و تحقیقاتی دانشگاه و تربیت دانشجویان کارشناسی و کارشناسی ارشد آنان، و دیگر مؤسسات آموزش عالی کشور مشارکت دارند. همچنین در این دانشگاه، در زمینه آموزش سایر رشته‌های مرتبط با بیماریها و اختلالات روانی، در حوزه‌های روانشناسی، رواندرمانی، روانپرستاری و توانبخشی آنها و جذب بهترین متخصصین این رشته‌ها در عضویت هیئت علمی دانشگاه، گامهای مؤثری در افزایش کیفیت آموزشها ارائه شده به دانشجویان و ارتقاء خدمات درمانی در مراکز تحت نظارت خود برداشته است. امید آنکه این ویژه نامه بتواند گامی در جهت پیشبرد تشخیص درمان و توانبخشی در بیماریها و اختلالات روانی و انعکاس فعالیت‌های پژوهشی آن با استعانت از پروردگار یکتای توانا باشد و واقعیت، ضرورت و اهمیت روانپزشکی را درحد استطاعت صفحات محدود این ویژه نامه بازتاب دهد. دکتر فربد فدایی – سردبیر افتخاری ویژه نامه روانپزشکی

Title: مقایسه تأثیر ریسپریدون با هالوپریدول بر نشانه‌های عمومی ‌اسکیزوفرنیا

http://rehabilitationj.uswr.ac.ir/article-1-122-fa.html

هدف: بررسی‌های انجام شده نشان داده است که ریسپریدون نسبت به هالوپریدول علائم عمومی را در بیماران مبتلا به اسکیزوفرنیا بیشتر کاهش می‌دهد. از آنجا که تأثیر این دارو در جمعیت ایرانی کمتر مورد تحقیق قرار گرفته است، بر آن شدیم تا به مقایسه تأثیر ریسپریدون (ساخت ایران) با هالوپریدول بر روی علائم عمومی اسکیزوفرنیا بپردازیم. روش بررسی: در یک مطالعه تجربی (Experimental) با طرح پیش آزمون–پس آزمون، 66 نفر از بیماران مرد اسکیزوفرنیک مطابق معیارهای (DSM-IV-TR)، بستری در مرکز روانپزشکی رازی، با انتخاب هدفمند، مورد مطالعه قرار گرفتند. پس از یک دوره دو هفته‌ای‌قطع دارو، بیماران به صورت تصادفی در دو گروه‌هالوپریدول (حداکثر 15 میلی‌گرم در روز) و ریسپریدون (حداکثر 6 میلی‌گرم در روز) قرارگرفتند. برای هر بیمار نمره کل زیر مقیاس (Subscale) عمومی پرسشنامه‌های مقیاس روانپزشکی مختصر (BPRS) و مقیاس بررسی عوارض خارج هرمی(SAS) در اندازه‌گیری پایه و 8 هفته پس از شروع درمان، به صورت هفتگی مورد بررسی قرارگرفت. نتایج درمانی توسط آزمونهای تی برای نمونه‌های جفتی و نمونه‌های غیر وابسته و آزمون تحلیل واریانس با اندازه‌گیری مکرر مورد تجزیه و تحلیل قرارگرفت. یافته‌ها: بین میانگین علائم عمومی در اندازه‌گیری پایه و در خلال درمان تا هفته پنجم بین دو گروه هالوپریدول و ریسپریدون اختلاف معنی‌داری وجود نداشت (P>0/05)، اما از هفته ششم تا انتهای طول درمان، میانگین علائم عمومی در گروه ریسپریدون به میزان معنی‌داری از گروه هالوپریدول کمتر بود (P<0/05). هردو دارو در طی تجویز باعث کاهش میانگین علائم عمومی بودند ولی این کاهش بین دو گروه اختلاف معنی‌داری داشت. همچنین شدت عوارض جانبی در تمام طول درمان در گروه ریسپریدون به میزان معنی‌داری نسبت به گروه هالوپریدول کمتر بود (P<0.05). نتیجه‌گیری: تجویز هالوپریدول و ریسپریدون ساخت ایران موجب کاهش معنی‌دار علائم عمومی در بیماران اسکیزوفرنیک ایرانی می‌شود که این کاهش در مورد ریسپریدون به میزان قابل توجهی بیشتر است. بعلاوه عوارض جانبی کمتری نیز دارد.

Title: دلایل تجویز درمان با تشنج الکتریکی در مرکز آموزشی درمانی روا‌ن‌پزشکی رازی

http://rehabilitationj.uswr.ac.ir/article-1-124-fa.html

هدف: درمان با تشنج الکتریکی اگر چه یکی از درمانهای مفید و مؤثر روانپزشکی است، ولی بدلیل تهاجمی بودن و عوارض محتمل آن، می‌بایست در موقعیتهایی که پژوهش‌های قبلی و کتب مرجع آنرا مؤثر دانسته‌اند بکار رود. این پژوهش با هدف تعیین دلایل تجویز تشنج الکتریکی در مرکز روانپزشکی رازی صورت گرفت. روش بررسی: در این مطالعه توصیفی بیمارانی که از تاریخ 1382/10/01 تا تاریخ 1383/12/30 در مرکز درمانی روانپزشکی رازی بستری شده بودند مورد مطالعه قرار گرفتند. گروه نمونه، بیمارانی بودند که بوسیله تشنج الکتریکی مداوا شده بودند. نمونه‌گیری به روش تمام شمار انجام شد. ابزار گرد‌آوری اطلاعات، فرم پرسشنامه محقق ساخته‌ای بود که توسط پزشک تجویزکننده تشنج الکتریکی تکمیل می‌گردید و تشخیص بیماری و دلیل تجویز تشنج الکتریکی و نیز سن و جنس بیماران را مورد سئوال قرار می‌داد. ‌داده‌های بدست آمده با استفاده از تستهای همبستگی و آزمون مجذور خی مورد تجزیه و تحلیل قرار گرفتند. یافته‌ها: شایعترین اختلالات تشخیص داده شده که در مرکز رازی بوسیله تشنج الکتریکی درمان شده عبارت بودند از: 1- اسکیزوفرنیا 2- اختلال دوقطبی 3- اختلال اسکیزوافکتیو. شایعترین دلایل تجویز تشنج الکتریکی در مرکز روانپزشکی رازی نیز عبارت بودند از: 1- مقاومت به درمان 2- شدید و حاد بودن علائم سایکوتیک و 3- وجود علائم کاتاتونیا و موتیزم. همچنین در این پژوهش مشخص شد که سن و جنس تأثیری بر تجویز تشنج الکتریکی در بیماران مرکز رازی نداشته است (P>0/05). نتیجه‌گیری: با توجه به اهمیت، مفید و مؤثر بودن تشنج الکتریکی از یک سو و نگرانیهای موجود پیرامون استفاده از این شیوه درمانی در موقعیتهایی که مورد تأیید کتب مرجع و پژوهش‌های قبلی نیستند، بنظر می‌رسد لازم است بر آموزش این نوع از درمان به دستیاران روانپزشکی و گروه‌های وابسته تأکید بیشتری شود و در دوره‌های بازآموزی روانپزشکان نیز جلساتی در مورد تشنج الکتریکی باشد.

Title: مقایسه انگ بیماری روانی در خانواده‌های بیماران مبتلا به اسکیزوفرنیا و اختلال افسردگی عمده بدون خصایص روانپریشی

http://rehabilitationj.uswr.ac.ir/article-1-125-fa.html

هدف: این مطالعه، با هدف مقایسه انگ بیماری روانی و عوامل مرتبط با آن، در خانواده‌های بیماران مبتلا به اسکیزوفرنیا و اختلال افسردگی عمده بدون خصایص روانپریشی، مطابق معیارهای DSM – IV – TR انجام شد. روش بررسی: مطالعه مقطعی- مقایسه‌ای حاضر با استفاده از روش نمونه گیری موارد در دسترس درمانگاه و بیمارستان روانپزشکی رازی و با انتخاب 40 نمونه از هر گروه اجرا گردید. ابزار اصلی جمع آوری داده‌ها، پرسشنامه 34 سئوالی خوداجرا بود، که در مطالعات پیشین دارای اعتبار شناخته شده و جهت بررسی انگ زدن طراحی شده است. یافته‌ها: نتایج مطالعه نشان داد که 45 درصد از افراد گروه اسکیزوفرنیا و 32/5 درصد از افراد گروه افسردگی عمده بدون خصایص روانپریشی، مورد تبعیض و تمسخر قرار گرفته‌اند. در گروه اسکیزوفرنیا بین مدت بیماری فرد، دفعات بستری شدن او و مورد تبعیض و تمسخر قرار گرفتن، رابطه آماری معنادار وجود داشت (P<0/05). همچنین در گروه اسکیزوفرنیا بین تشخیص بیماری و مخفی نگه داشتن بیماری فرد توسط خانواده ارتباط آماری معنادار بود (P<0/05). نتیجه‌گیری: نیمی از خانواده‌های بیماران روانی مورد تمسخر قرار می‌گیرند، و به علت انگ بیماری روانی احساس ناراحتی و شرم می‌کنند. میزان انگ با نوع بیماری ارتباط دارد. استفاده از آموزش روانی خانواده‌ها و نیز آموزش در سطح جامعه در بالا بردن سطح آگاهی و کاهش انگ بیماری روانی می‌توانند مؤثر باشد.

Title: بررسی میزان شیوع اختلالات رفتاری در دانش‌آموزان دوره ابتدایی استان ایلام

http://rehabilitationj.uswr.ac.ir/article-1-126-fa.html

هدف: اختلالات رفتاری طیف وسیعی از مشکلات دوران کودکی را تشکیل می‌دهد که شیوع آن در مناطق مختلف ایران و جهان دارای نوسانات بسیاری است. پژوهش حاضر به بررسی میزان شیوع اختلالات رفتاری در دانش‌آموزان دوره ابتدایی استان ایلام می پردازد. روش بررسی: این پژوهش یک مطالعه توصیفی و مقطعی است که جامعه آماری آن را کلیه دانش‌آموزان مدارس ابتدایی استان ایلام در سال تحصیلی 84-1383 تشکیل می دادند. حجم نمونه پژوهش حاضر 1546 دانش‌آموز بود که با استفاده از فرمول حجم نمونه و براساس شیوه نمونه‌گیری طبقه‌ای برحسب جنسیت، منطقه و محل زندگی و پایه تحصیلی و به روش نمونه‌گیری خوشه‌ای چند مرحله‌ای جهت تکمیل پرسشنامه انتخاب شدند. از پرسشنامه راتر فرم معلم به عنوان ابزار غربالگری و فرم مصاحبه تشخیصی روانپزشکی برای دانش‌آموزانی که در آزمون راتر نمره بالاتر از نقطه برش داشتند، جهت تأیید تشخیص استفاده گردید. برای تحلیل داده‌ها آزمون خی دو مورد استفاده قرار گرفت یافته‌ها: نتایج نشان داد که میزان شیوع اختلالات رفتاری در دانش‌آموزان دوره ابتدایی استان ایلام 2/10 درصد می‌باشد. میزان شیوع در پسران بیشتر از دختران، در مناطق شهری بیشتر از مناطق روستایی و در پایه تحصیلی سوم بیشتر از سایر پایه‌های تحصیلی است. همچنین نتایج میزان شیوع برحسب زیرگروه‌های طبقات تشخیصی اختلالات رفتاری آزمون راتر نشان داد که اختلال ناسازگاری اجتماعی بیشترین و اختلال رفتارهای ضداجتماعی کمترین میزان شیوع را داشتند و در بین عوامل جمعیت‌شناختی، تحصیلات والدین، طلاق والدین و سابقه بیماری روانی در خانواده با بروز اختلالات رفتاری در کودکان رابطه معناداری داشتند (P>0/05). نتیجه‌گیری: میزان شیوع اختلالات رفتاری در استان ایلام در مقایسه با سایر پژوهشهای انجام گرفته در داخل و خارج از کشور در دامنه متوسط قرار دارد.

Title: مدلیابی خود اثرمندی جسمانی در افسردگی نوجوانان بر مبنای مدل عاملیتی بندورا از افسردگی

http://rehabilitationj.uswr.ac.ir/article-1-127-fa.html

هدف: این پژوهش با هدف بررسی و مدلیابی تأثیر مستقیم و غیرمستقیم خوداثرمندی جسمانی بر افسردگی نوجوانان انجام شده است. روش بررسی: آزمودنیهای این پژوهش توصیفی – مقطعی، 946 نفر از دانش‌آموزان مقطع دبیرستان و پیش دانشگاهی شهر تهران می‌باشند که به روش نمونه‌گیری خوشه‌ای چند مرحله‌ای- تصادفی انتخاب شده اند. ابزار گردآوری اطلاعات عبارتند از: سیاهه افسردگی کودک (CDI)، پرسشنامه خوداثرمندی جسمانی، پرسشنامه اجتناب اجتماعی و پرسشنامه طرد همسالان. پس از تکمیل پرسشنامه‌ها توسط نمونه‌های پژوهش، بــرای تجزیه تحلیل داده‌ها از نرم افزار کامپیوتری لیزرل و روش مدلیابی معادلات ساختاری استفاده شد. روش مدلیابی ساختاری اساساً برای مشخص کردن پدیده مورد مطالعه در قالب متغیرهای علت و معلولی استفاده می‌گردد. یافته‌ها: از طریق روش تحلیل عاملی، دو عامل خود اثرمندی مهارت و تسلط جسمانی و خوداثرمندی ارائه مهارتهای جسمانی مشخص شده و مورد تحلیل قرار گرفتند. نتایج (RMR=0/079, RMSEA=0/047, NFI=0/95, CFI=0/97, GFI=0/96) نشان داد که تأثیر مستقیم خوداثرمندی مهارت و تسلط جسمانی بر افسردگی معنی‌دار نمی‌باشد (P>0/05). اما تأثیر غیرمستقیم آن معنی‌دار است (P<0/05). همچنین تأثیر مستقیم خوداثرمندی در ارائه مهارتهای جسمانی بر افسردگی و نیز تأثیر غیرمستقیم آن معنی‌دار می‌باشد (P<0/05). نتیجه‌گیری: خوداثرمندی ارائه مهارتهای جسمانی و کسب ارزیابی مثبت اجتماعی به طور مستقیم و همچنین به طور غیرمستقیم از طریق اجتناب اجتماعی و طرد همسالان بر افسردگی نوجوانان مؤثر است. خوداثرمندی مهارت و تسلط جسمانی بر افسردگی نوجوانان تأثیر مستقیم نداشته و از طریق اجتناب اجتماعی و طرد همسالان بر افسردگی نوجوانان مؤثر است.

Title: تعیین اثربخشی نوروفیدبک بر عملکرد هوشی کودکان مبتلا به اختلال بیش‌فعالی/نقص توجه

http://rehabilitationj.uswr.ac.ir/article-1-129-fa.html

هدف: این پژوهش به منظور بررسی اثربخشی روش نوروفیدبک و مقایسه آن با ریتالین و درمان ترکیبی نوروفیدبک و ریتالین توأم در کاهش علائم اختلال بیش‌فعالی / کمبود توجه انجام شده، یعنی اثربخشی این روشهای درمانی را بر افزایش نمرات هوشی آزمودنیها مد نظر قرار داده است. روش بررسی: در این پژوهش شبه تجربی که به شکل (Case series) انجام شد، تعداد 16 نفر آزمودنی مبتلا به اختلال بیش‌فعالی – کمبود توجه نوع مرکب که به صورت هدفمند انتخاب شده بودند، در 4 گروه مختلف به مدت 10 هفته تحت درمان با هر یک از سه رویکرد فوق قرارگرفتند. گروه چهارم هیچ درمانی نگرفتند. یافته‌ها: نتایج نشان داد که درمان ترکیبی نوروفیدبک و ریتالین در هر 4 آزمودنی موجب افزایش هوشبهر شده است. درمان با ریتالین به تنهایی نیز در هر 4 آزمودنی موجب افزایش نمرات هوشی گردید، اما این میزان در برخی موارد خیلی قابل توجه نبود. روش درمانی نوروفیدبک نیز در 3 نفر از آزمودنیها موجب افزایش نمره هوشبهر گردید. در گروه چهارم تغییراتی حاصل نشد. نتیجه‌گیری: اثربخشی درمان ترکیبی نوروفیدبک و ریتالین در افزایش نمرات کودکان مبتلا به اختلال بیش‌فعالی/نقص توجه در آزمون هوشی تجدید نظر شده وکسلر کودکان بیشتر از هریک از دو روش دیگر به تنهایی است.

Title: مقایسه سازگاری اجتماعی مادران دختران عقب‌مانده ذهنی آموزش‌پذیر با مادران دختران عادی شهر کاشان

http://rehabilitationj.uswr.ac.ir/article-1-128-fa.html

هدف: در این تحقیق سازگاری اجتماعی مادران دختران عقب‌مانده ذهنی آموزش پذیر با سازگاری اجتماعی مادران دختران عادی مقایسه شده است. هدف از این مطالعه پاسخ به این پرسش بود که آیا حضور کودک عقب‌مانده ذهنی بر سازگاری اجتماعی مادران تأثیر منفی دارد یا نه؟ روش بررسی: این پژوهش یک مطالعه مورد- شاهدی از نوع تحلیلی است. نمونه‌های این تحقیق شامل 80 نفر، 40 مادر دارای فرزند دختر عقب‌مانده ذهنی با انتخاب تصادفی از مدارس استثنایی و 40 مادر دارای فرزند دختر عادی با انتخاب تصادفی از بین مادرانی که با گروه مورد همتاسازی شده بودند، می‌باشد. در این مطالعه از مقیاس سازگاری اجتماعی ویزمن وپی‌کل (1974) استفاده شد. این مقیاس یک مصاحبه نیمه سازمان یافته است که سازگاری اجتماعی رادرهفت نقش یا حوزه اصلی بررسی می‌کند. این نقشها شامل: سازگاری در شغل، فعالیتهای اجتماعی و فوق برنامه، روابط خویشاوندی، روابط زناشویی، نقش والدینی، روابط خانوادگی و وضعیت اقتصادی می‌باشد. یافته‌ها: نتایج نشان داد که بین سازگاری اجتماعی مادران دختران عقب‌مانده ذهنی با سازگاری اجتماعی مادران دختران عادی تفاوت معنی‌داری وجود دارد (P<0/01). آنالیز واریانس و همبستگی نشان داد که بین سازگاری اجتماعی مادران دختران عقب‌مانده ذهنی با متغیرهای دموگرافیک سن کودک، سن مادر، میزان تحصیلات،‌ طول مدت ازدواج، تعداد فرزندان وترتیب تولد فرزند عقب‌مانده ذهنی ارتباط معنی‌داری وجود ندارد (P<0/01). نتیجه‌گیری: با توجه به نتایج حاصله به نظر می‌رسد در مجموع مادران دارای دختر عقب‌مانده ذهنی نسبت به مادران دختران عادی سازگاری اجتماعی کمتری دارند.

Title: نقش تازه‌جویی و هیجان طلبی در سوء مصرف و وابستگی به مواد شبه‌افیونی

http://rehabilitationj.uswr.ac.ir/article-1-130-fa.html

هدف: از عوامل مؤثر در گرایش به سوء مصرف مواد، عوامل شخصیتی است. در این میان مؤلفه تازه‌جویی و هیجان‌طلبی (یکی از مؤلفه‌های مزاجی شخصیت) بطور بارزی با استعداد ابتلا به سوء مصرف مواد همراه است. هدف از پژوهش حاضر بررسی دقیق‌تر ارتباط بین سوء مصرف و وابستگی به مواد شبه افیونی با مؤلفه مزاجی تازه‌جویی می‌باشد. روش بررسی: این پژوهش به روش تحلیلی مورد – شاهدی برروی یکصد نفر از مراجعین به دو مرکز ترک اعتیاد در شهر تهران که بطور تصادفی انتخاب شده بودند انجام شد. گروه مورد برمبنای ملاکهای تشخیصی D‏SM–IV–TR به سوء مصرف یا وابستگی به مواد شبه افیونی مبتلا بودند. گروه شاهد نیز از بین آن دسته از همراهان مراجعین که مطابق همتاسازی با گروه مورد مشابهت داشتند و براساس ملاکهای فوق، سابقه هیچگونه سوء مصرف و یا وابستگی به مواد را نداشتند، بطور تصادفی انتخاب شدند. در مورد همه آزمودنیها به ترتیب پرسشنامه ویژگیهای دموگرافیک و مصاحبه نیمه ساختار یافته سوء مصرف یا وابستگی مواد برمبنای DSM–IV–TR و سپس پرسشنامه مقیاس محرک خواهی «هیجان‌طلبی» زاکرمن اجرا گردید. یافته‌ها: افراد مبتلا به سوء مصرف و وابستگی به مواد اپیوئیدی نسبت به گروه شاهد، میانگین نمرات تازه‌جویی یا هیجان‌طلبی بالاتری را نشان می‌دادند که این تفاوت معنی دار بود (P<0/05). بین سن شروع اولین سوء مصرف مواد و مؤلفه تازه‌جویی، همبستگی منفی و معنی‌دار وجود داشت (P<0/05). همچنین ارتباطی معنی دار بین سابقه سوء مصرف مواد به شکل تزریقی با میزان بالاتر مؤلفه تازه‌جویی مشاهده شد. نتیجه‌گیری: با توجه به رابطه بین سوء مصرف مواد و مؤلفه مزاجی تازه‌جویی و نیز قابلیت سنجش این مؤلفه با یک آزمون روانشناختی معتبر و قابل اجرا، به راحتی می‌توان افراد پرخطر را در سنین نوجوانی مشخص کرده و اقدامات پیشگیری اولیه را بطور مؤثرتری به اجرا گذاشت.

Title: کارکرد خانواده در بیماران دچار اختلال هویت جنسی

http://rehabilitationj.uswr.ac.ir/article-1-131-fa.html

هدف: اختلال هویت جنسی یک وضعیت نسبتاً نادر است که در آن افراد، هویت جنسی خود را با آنچه ظاهر آنها نشان می‌دهد نامتجانس احساس می‌کنند. از عوامل مهم مرتبط با آن خانواده را بوده که احتمالاً در سبب شناسی و در عین حال نگهداری آن مؤثر است. به این ترتیب بررسی رابطه کارکرد خانواده با این اختلال به خصوص در کشور ایران که هدف این مطالعه نیز می‌باشد، می‌تواند در بالا بردن کیفیت کمک به بیماران مبتلا مفید واقع شود. روش بررسی: این پژوهش به روش مقطعی مقایسه‌ای و از نوع مورد – شاهدی و با نمونه‌گیری در دسترس انجام شد که در آن تعداد 37 نفر از مراجعه‌کنند‌گان به سازمان پزشکی قانونی که تشخیص اختلال هویت جنسی، توسط کمیسیون روانپزشکی در آنها محرز شده بود، پرسش‌نامه‌های اطلاعات فردی و ابزار سنجش خانواده که ابزاری برای بررسی کارکرد خانواده است را تکمیل نمودند (گروه نمونه) و با روش همسان سازی 37 نفر از افراد در دسترس که با ارزیابی روانپزشک فاقد هرگونه اختلال روانپزشکی بودند نیز به عنوان گروه مقایسه پرسش‌نامه‌ها را تکمیل نمودند. داده‌ها با استفاده از آزمون تی برای گروه‌های مستقل مورد تجزیه و تحلیل قرار گرفت. یافته‌ها: نتایج نشان داد که «کارکرد خانواده» (P=0/034) و همچنین ابعاد کارکردی «آمیختگی عاطفی» (P=0/03)، «کنترل رفتار»(P=0/021) و «پاسخگویی عاطفی» (P=0/009) در گروه نمونه نسبت به گروه مقایسه دارای اختلال معنادار و از کارایی پایین‌تری برخوردار بود، در حالی که این تفاوت در مورد ابعاد کارکردی «نقش‌ها» (P=0/15)، «حل مسئله» (P=0/16) و «ارتباط» (P=0/066) به طور معنادار به چشم نمی‌خورد. نتیجه‌گیری: با توجه به یافته‌های پژوهش وجود اختلال هویت جنسی در افرادی که دچار مسائل و مشکلات عاطفی، احساسی و هیجانی بیشتری در خانواده هستند محتمل‌تر و کمک گسترده به این افراد از طریق مداخلات خانوادگی می‌تواند مؤثر واقع شود. همچنین آگاهی دادن به خانواده‌ها در مورد نیازهای عاطفی و تربیتی فرزندان، احتمال بروز این اختلال را کاهش می‌دهد.

Title: فرسودگی شغلی و عوامل مرتبط با آن در کارمندان بیمارستان روانپزشکی رازی

http://rehabilitationj.uswr.ac.ir/article-1-132-fa.html

هدف: هدف این مطالعه بررسی میزان فرسودگی شغلی و ابعاد مختلف آن در کارکنان درمانی و اداری بیمارستان روانپزشکی رازی تهران و ارتباط آن با متغیرهای جمعیت شناختی و سایر عوامل مرتبط بود. روش بررسی: این مطالعه یک پژوهش تحلیلی مقطعی بود. بر اساس نمونه‌گیری طبقه‌ای- تصادفی، تعداد 250 نفر به عنوان حجم نمونه نهایی متناسب با تعداد کارمندان واحدها انتخاب شدند. به علت عدم همکاری و نقص در تکمیل اطلاعات، مطالعه نهایی بر روی 198 نفر انجام گرفت. برای تمام افراد پرسشنامه فرسودگی شغلی مازلاخ و پرسشنامه‌ جمعیت شناختی تکمیل شد. یافته‌ها: میانگین نمره فرسودگی شغلی در کل جمعیت مورد مطالعه 29/06 و در حد خفیف بود. فرسودگی شغلی در کارکنان درمانی به نسبت اداری، نمره بالاتری را به خود اختصاص داده بود و این اختلاف از نظر آماری معنی‌دار بود. اختلاف بین میانگین نمره مازلاخ بین کارمندان درمانی و اداری بیمارستان در زمینه‌های خستگی هیجانی و احساس کفایت شخصی از نظر آماری معنی‌دار بود، ولی در زمینه مسخ شخصیت ارتباط معنی‌داری وجود نداشت. فرسودگی شغلی با جنسیت و ساعات کار کارمندان در هفته، دارای ارتباط معنی‌دار ولی با سن کارمندان و وضعیت تأهل و میزان متوسط درآمد ماهیانه آنها فاقد ارتباط معنی‌دار بود. نتیجه‌گیری: فرسودگی شغلی (هرچند خفیف) به نحو قابل ملاحظه‌ای در کارکنان مرکز روانپزشکی رازی وجود دارد. این وضعیت شکل جدی تری در کارکنان درمانی بیمارستان دارد. امیدواریم نتایج به دست آمده از این مطالعه بتواند برای برنامه‌ریزی بهتر در استفاده از نیروهای تخصصی و ارائه خدمات بهتر به بیماران روانپزشکی مفید باشد.

Title: بررسی نقش آموزش مهارت‌های ارتباطی و حل مسئله اجتماعی بر عزت نفس و هوش‌بهر دانش‌آموزان

http://rehabilitationj.uswr.ac.ir/article-1-133-fa.html

هدف: هدف از این پژوهش بررسی نقش آموزش مهارتهای ارتباطی و حل مسئله اجتماعی بر عزت نفس و هوشبهر دانش‌آموزان مقطع سوم دبستان است. روش بررسی: برای انجام این مطالعه، روش تجربی پیش‌آزمون- پس‌آزمون انتخاب شد. چهل و هشت دانش‌آموز سوم ابتدایی به طور تصادفی به دو گروه مورد و شاهد تقسیم شدند. گروه مورد طی برنامه مهارتهای ارتباطی و حل مسئله به مدت 15 جلسه تحت آموزش قرار گرفتند. برای جمع آوری داده‌ها از پرسشنامه‌های هوش‌آزمای بازنگری شده وکسلر کودکان و اعتمادبه نفس پاپ و مک‌هیل استفاده گردید. در تحلیل داده‌ها، آزمون‌ تی به کار گرفته شد. یافته‌ها: تحلیل‌آماری پیش‌آزمون و پس‌آزمون نشان داد که آموزش به طور معنی‌داری نمرات هوش را در میان گروه مورد بهبود بخشیده است (P<0/05). تحلیل‌ آماری همچنین از بهبود معنی‌دار اعتماد به نفس در این گروه حکایت داشت. نتیجه‌گیری: یافته‌ها، بیانگر نقش و اثر مثبت آموزش مهارت‌های ارتباطی و حل مسئله اجتماعی بر اعتماد به نفس و هوشبهر می‌باشد.

Title: بررسی تأثیر خدمات روانپرستاری مراقبت در منزل بر وضعیت روانی بیماران اسکیزوفرنیک

http://rehabilitationj.uswr.ac.ir/article-1-134-fa.html

هدف: افزایش هزینه‌های بستری در بیمارستان، دوری از خانواده و خطر ابتلا به عفونتهای بیمارستانی منجر گردیده است تا روز به روز خدمات مراقبت در منزل مورد استقبال بیشتری قرارگیرد. از طرفی در بیماریهای روانی طولانی مدت از جمله اسکیزوفرنیا به دلیل اینکه بهبودی در آینده نزدیک متصور نمی‌باشد لذا ادامه مراقبت‌ها باید در منزل انجام پذیرد. هدف این پژوهش بررسی تأثیر مراقبت در منزل بر وضعیت روانی بیماران اسکیزوفرنیک با سیر طولانی مدت بود. روش بررسی: این پژوهش از نوع تجربی بوده است که در آن 48 نفرمددجوی اسکیزوفرنیک بطور تصادفی در دو گروه آزمون وکنترل جای گرفتند(هر گروه 24 نفر). مداخله پژوهش برای گروه آزمون پس از ترخیص از بیمارستان شامل مراقبت‌های پرستاری در منزل بود و برای گروه کنترل این مداخله انجام نگردید و روش جاری شامل مراجعه مددجو به درمانگاه روانی جهت ادامه درمانها انجام شد. دو گروه برای بررسی وضعیت روانی در هنگام بستری، هنگام ترخیص و سه ماه پس از ترخیص با پرسشنامه دموگرافیک و مقیاس مختصر روانپزشکی B‏PRS مقایسه شدند. برای تجزیه تحلیل داده‌ها از جداول توزیع فراوانی مطلق و نسبی و همچنین برای بررسی روابط بین متغیرها از آزمون آماری تی مستقل و آزمون مجذور کای در سطح معنی‌داری 95 درصد استفاده گردید. یافته‌ها: نتایج نشان داد که وضعیت روانی مددجویانیکه خدمات مراقبت در منزل دریافت می‌کردند بهتر از گروهی بود که این مراقبت را دریافت ننمودند و این تفاوت از نظر آماری معنی‌دار بود (P=0/007). نتیجه‌گیری: خدمات مراقبت پرستاری در منزل برای بیماران مبتلا به اسکیزوفرنیا مؤثر می‌باشد و می‌تواند به بهبود وضعیت روانی آنها منجر گردد.

Title: مختصات روان‌سنجی پرسشنامه افسردگی بک -2 در یک نمونه بزرگ مبتلایان به اختلال افسردگی اساسی

http://rehabilitationj.uswr.ac.ir/article-1-135-fa.html

هدف: مطالعه حاضر به گزارش مختصات روانسنجی پرسشنامه افسردگی بک – 2 در ایران اختصاص دارد. روش بررسی: در یک مطالعه توصیفی کاربردی از نوع روانسنجی و اعتباریابی آزمون، 354 تن از افرادی که تشخیص افسردگی اساسی را دریافت داشته و در زمان مطالعه در دوره بهبودی نسبی به سر می‌بردند، با انتخاب هدفمند، به منظور تعیین درجه افسردگی و سنجش اثرات کارآزمایی‌های بالینی مورد ارزیابی قرار گرفتند. این آزمودنی‌ها از طریق یک فراخوان عمومی از مراکز روانشناسی و رواندرمانی و معرفی به مراکز اجرای طرح، پس از شرکت در مصاحبه بالینی ساختار یافته برای اختلالات DSM-IV و با رعایت ملاک‌های ورود و خروج مطالعه در طرح پژوهش قرار گرفتند. علاوه بر انجام مصاحبه بالینی، آزمودنی‌ها به پرسشنامه افسردگی بک-2 و مقیاس افسردگی پرسشنامه نشانگان مختصر پاسخ دادند. این مقاله اختصاص به گزارش نتایج مربوط به اجرای پرسشنامه افسردگی بک – II دارد. یافته‌ها: ‌نتایج حاصل از تحلیل عاملی، سنجش اعتبار و روایی پرسشنامه افسردگی بک-2 حکایت از کارآیی آن داشت. نتیجه گیری: پرسشنامه افسردگی بک – II در مسیر آزمون مختصات روانسنجی نشان داد که به طور پایایی برای تشخیص و سنجش شدت افسردگی قبل و بعد از درمان و در دفعات متعدد کاربرد دارد. نتایج تحلیل عاملی و سنجش روایی که در این مطالعه حاصل شد حکایت از تناسب آن برای ارزشیابی نتایج هر نوع کارآزمایی بالینی دارد. بویژه که می‌توان با توجه به عوامل سه گانه افسردگی مشخص کرد که تغییرات در چه ابعادی رخ می‌دهد.
